# A study on the efficiency and spatial correlation of health resource allocation in Chinese hospitals

**DOI:** 10.1371/journal.pone.0356197

**Published:** 2026-09-08

**Authors:** Yan Zhao, Guang Song, Weilong Zhao, Ting Min, Yaxing Nan

**Affiliations:** 1 School of Health Management, Gansu University of Chinese Medicine, Lanzhou, Gansu, China; 2 School of Health Management, Bailie Vocational College, Zhangye, Gansu, China; 3 Department of Health Policy and Management, School of Public Health, Lanzhou University, Lanzhou, China; University of Economics Ho Chi Minh City, VIET NAM

## Abstract

**Background:**

This study quantitatively evaluated the efficiency of hospital healthcare resource allocation across 31 provinces in China from 2019 to 2023, with the aim of providing evidence-based decision support for improving resource utilization efficiency and promoting the sustainable development of medical institutions.

**Methods:**

A three-stage Data Envelopment Analysis (DEA)–Malmquist model was employed to estimate both static and dynamic efficiency in hospital healthcare resource allocation. Spatial autocorrelation of efficiency was examined using Moran’s I to identify regional spatial dependence patterns.

**Results:**

The average comprehensive efficiency of healthcare resource allocation in Chinese hospitals was below 1, indicating substantial room for improvement in overall efficiency, though an overall upward trend was observed during the study period. After controlling for environmental factors, the mean comprehensive efficiency declined from 0.858 to 0.827, suggesting that external environmental conditions influenced actual efficiency to some extent. Dynamic analysis indicated that technical efficiency was the primary constraint on total factor productivity growth. In regional comparisons, after accounting for environmental factors, the efficiency ranking shifted from the original pattern of eastern > central > western to a temporary order of central > eastern > western. Spatial analysis indicated a significant positive spatial correlation in resource allocation efficiency. A stable high-efficiency cluster persisted in the region south of the Yangtze River, while low-efficiency clusters had largely dissipated by 2023, reflecting a convergence trend of provincial efficiency levels toward the efficiency frontier.

**Conclusions:**

Hospital efficiency in China exhibits a distinct east-to-west gradient. However, after adjusting for environmental heterogeneity, the central region temporarily outperformed the eastern region, highlighting the confounding effects of external factors. Robust spatial spillover effects were observed. Policy interventions should therefore prioritize targeted efficiency improvements in low-efficiency cold-spot provinces over mere input expansion. The bias-corrected efficiency scores and spatial clustering patterns provide a validated benchmark for national and regional health resource planning.

## Introduction

Hospital efficiency is a critical lever for achieving universal health coverage, particularly in aging societies [1–3]. Defined as the ratio of observed to maximum attainable output for a given set of inputs [4,5]. Efficiency is intrinsically linked to the spatial organization of healthcare delivery. Spatial agglomeration—the non-random clustering of medical facilities and related industries [6]-can foster local knowledge spillovers and enhance patient accessibility [7]. Together, efficiency and agglomeration shape how limited health resources are translated into equitable and effective lifelong services, spanning disease prevention, health promotion, treatment, and chronic disease management [8,9].

Following the 2009 healthcare reform, China expanded its health system capacity through fiscal subsidies and workforce recruitment [10]. From 2012 to 2023, the number of licensed medical institutions increased by 11%, from 0.95 to 1.07 million [11]. However, investments in medical equipment have yielded significant efficiency gains only in highly urbanized regions, leaving less urbanized areas with persistently lower performance [12,13]. Against the backdrop of a growing aging population and rising chronic disease burdens, optimizing efficiency and spatial allocation has become increasingly urgent.

This policy evolution provides a pertinent context for re-examining hospital efficiency. While Zhou et al. [14] identified equity gaps across specialized public health institutions, efficiency at the hospital level—where the majority of outpatient and inpatient care occurs—remains quantitatively underexplored. Building on this foundation, our study extends the equity lens to the production side, investigating how far hospital resources are from their maximum attainable output after controlling for environmental noise. This shift connects broad policy objectives with specific operational performance.

The inequitable distribution of health resources is a persistent global challenge [15]. Internationally, numerous studies employ Data Envelopment Analysis (DEA) to assess health system performance. For instance, Sajadi used the Malmquist index to evaluate health resource utilization efficiency in Iran [16], while Barouni et al. applied the Gini coefficient to assess equity in primary health care budget allocation across Iranian provinces [17]. Other research has examined epidemiological trends and resource utilization in Japan [18], including the specialty and practice distribution of physicians [19]. In the Chinese context, research on hospital-based resources clusters around three analytical pillars, adapted here for hospital-level analysis. First, a macro-efficiency strand evaluates welfare generated per unit of Gross Domestic Product (GDP) allocated to health expenditure [20] and assesses systemic shifts following policy reforms such as the 2017 national medical-insurance global budgeting [21]. Second, studies examine regional disparities by pairing individual and allocation efficiency metrics [22] and mapping the spatial evolution of resources and productivity [23]. Third, structural-efficiency research explores policy levers such as strategic purchasing, health information technology [24], and local pilot schemes, often evaluating system performance through the dual lens of equity and efficiency [25]. While several studies have employed three-stage DEA or spatial analysis individually, systematic evaluations that integrate bias-corrected dynamic efficiency (Malmquist) with spatial autocorrelation during the critical 2019–2023 transition period remain scarce. This study goes beyond mere methodological application by capturing the ‘V-shaped’ recovery of hospital productivity through a bias-correction lens, revealing how the COVID-19 pandemic and subsequent policy shifts fundamentally reshaped regional efficiency gradients in ways that conventional models might overlook. As a result, the geographic clustering of inefficiency across provinces and its modifiable environmental drivers remain empirically unclear [26]. Despite these valuable contributions, three critical gaps remain. First, no study has systematically evaluated hospital-level efficiency across the full 2019–2023 period that includes the COVID-19 shock, adaptation, and recovery phases. Second, conventional DEA models rarely distinguish true managerial performance from “environmental dividends” (e.g., regional GDP or infrastructure), potentially biasing efficiency estimates. Third, the spatial interdependence of hospital efficiency—such as spillovers from patient mobility or telemedicine networks—remains empirically unexplored.

To address this gaps, this study employs a three-stage DEA-Malmquist model with spatial autocorrelation analysis to provide bias-corrected efficiency estimates for hospital resource allocation across 31 Chinese provinces from 2019 to 2023. The study aims to provide empirical evidence and policy insights for improving the efficiency of hospital healthcare resource allocation and promoting equity in essential public health services.

## Methods

### Data sources and variables

Hospital-level panel data from 2019 to 2023 were obtained from three official publications of China's National Bureau of Statistics: China Health Statistics Yearbook, China Health-Care Statistics Yearbook, and China Statistical Yearbook. All monetary values were adjusted to 2019 constant prices using provincial GDP deflators. Consistent with the National Bureau of Statistics (NBS) regional classification scheme and common practice in hospital efficiency studies, the 31 provincial-level units were categorized into three regions: Eastern (n = 11), Central (n = 8), and Western (n = 12). This grouping preserves sufficient degrees of freedom for regional subgroup analysis.

Input and output variables were selected based on data availability across all provinces and years, as well as their established use in recent efficiency literature. Three inputs were chosen to reflect core hospital resources: the number of medical institutions, hospital beds, and health technicians. Two outputs captured primary hospital service volumes: outpatient visits and inpatient discharges. This set avoids the double-counting often associated with financial indicators. To account for external conditions, three environmental variables—per capita GDP, urbanization rate, and urban population density—were included for adjustment in the second-stage analysis [27,28]. Administrative boundary data were obtained from Natural Earth Data (https://www.naturalearthdata.com), a public domain dataset.

### Research methods

#### Three-stage DEA-Malmquist index model.

To achieve the research objective, this study employs an integrated framework comprising three distinct methodological stages: (1) Data Envelopment Analysis (DEA), specifically the Banker-Charnes-Cooper (BCC) model, to measure initial efficiency; (2) Stochastic Frontier Analysis (SFA) to adjust for environmental noise; and (3) the Malmquist Productivity Index to analyze dynamic changes in Total Factor Productivity (TFP). The comprehensive computational logic of this integrated framework is summarized in S1 Text and S1 Fig in S1 Appendix. Integrated Three‑stage DEA‑Malmquist‑Spatial Analysis Framework. Following these stages, the analysis ensures a bias-corrected estimation of hospital resource allocation efficiency across the 31 provincial units [29–36]. Stage 1: Initial Efficiency Evaluation. We apply the input-oriented Banker-Charnes-Cooper (BCC) model [37] to calculate initial efficiencies and slacks:


$$\begin{array}{c} {𝐱}_{𝐢𝐣}>0,\;{𝐲}_{𝐫𝐣}>0,\;𝐣=1,2...,\;𝐧,\;𝐢=1,\;2,\;...,\;𝐦,\;𝐫=1,\;2,\;...,\;𝐬. \end{array}$$ (1)

In this context, m and s represent, respectively, the numbers of input and output variables for each decision-making unit.

After introducing slack variables ${\text{s}}^{-}$, ${\text{s}}^{+}$ and non-Archimedean infinitesimals ϵ, and performing dual planning, the standard input-oriented BCC model is as follows:


$$\begin{array}{c} 𝐦𝐢𝐧\varphi_{0}=\theta_{0}-\epsilon \left(\sum_{𝐢}^{𝐦}{𝐒}_{𝐢}^{-}+\sum_{𝐫=1}^{𝐬}{𝐒}_{𝐫}^{+}\right), \end{array}$$ (2)

$$\begin{array}{c} {𝐒}_{.}{𝐭}_{.}\left\{ \begin{array}{c} \begin{array}{c} \&\sum_{𝐣-1}^{𝐧}\lambda_{𝐣}{𝐱}_{𝐢𝐣}+{𝐒}_{𝐢}^{-}=\theta_{0}{𝐗}_{𝐢𝐨}\\ \sum_{𝐣=1}^{𝐧}\lambda_{𝐣}{𝐲}_{𝐫𝐣}-{𝐒}_{𝐫}^{+}={𝐘}_{𝐫𝐨}\\ \&\sum_{𝐣=1}^{𝐧}\lambda_{𝐣}=1 \end{array}\\ {𝐒}_{𝐢}^{-},\;{𝐒}_{𝐫}^{+},\;\lambda_{𝐣}\geq 0,\;𝐣=1,\;2,\;...,𝐧. \end{array}\;\right. \end{array}$$ (3)

In these equations, $\varphi_{\text{0}}$ is the efficiency score, $\lambda_{j}$ represents the weighting coefficients, $S_{i}^{-}$ and $\sum_{j=1}^{n}\lambda_{j}y_{rj}-S_{r}^{+}$ are the slack variables for inputs and outputs, and ϵ is the non-Archimedean infinitesimal. The relationships among Technical Efficiency (TE), Pure Technical Efficiency (PTE), and Scale Efficiency (SE) are as follows.


$$\begin{array}{c} 𝐓𝐄=𝐏𝐓𝐄\times 𝐒𝐄. \end{array}$$ (4)

Stage 2: Environmental Adjustment. SFA regression is used to decompose slacks into environmental effects, random noise, and managerial inefficiency [35]:


$$\begin{array}{c} {𝐒}_{𝐧𝐢}=𝐟\;({𝐙}_{𝐧𝐢},\beta {)+{𝐯}_{𝐧𝐢}+\text{u}}_{𝐧𝐢},\;𝐢=1,\;2.\;𝐍,\;𝐧=1,\;2,\; \end{array}$$ (5)

Where $S_{ni}$ is the input slack, *(*$Z_{i}$*,* $\beta_{n}$*) is the deterministic feasible frontier function,* $Z_{i}$ *represents the environmental variables,* $\beta_{n}$ *is the parameter vector to be estimated,* $v_{ni}$ *is the random error, and* $u_{ni}$ *is the managerial inefficiency.* This mechanism is specifically designed to manage experimental biases and statistical errors by stripping away external confounding factors, ensuring that the final efficiency scores reflect true managerial performance. Based on the SFA results, the raw inputs are adjusted to ensure all units are compared under a standardized environment:


XniA=Xni+[𝐦𝐚𝐱(𝐟(𝐙𝐢;^β𝐧))−𝐟(𝐙𝐢;^β𝐧)]+ [𝐦𝐚𝐱(𝐯𝐧𝐢)−vni]. (6)

$$\begin{array}{c} \gamma =\frac{\sigma_{{𝐮}_{𝐧𝐢}}^{2}}{\sigma_{{𝐯}_{𝐧𝐢}}^{2}+\sigma_{{𝐮}_{𝐧𝐢}}^{2}} \end{array}$$ (7)

In equation (7),γ the ratio of management inefficiency variance to total variance.


$$\begin{array}{c} \text{E}(\text{u}|\epsilon )=\;\sigma *[\frac{\varphi (\frac{\lambda \epsilon }{\sigma })}{\Phi (\frac{\lambda \epsilon }{\sigma })}+\frac{\lambda \epsilon }{\sigma }].\; \end{array}$$ (8)

In equation (8) $\sigma *=\frac{\sigma_{𝐮}\sigma_{𝐯}}{\sigma }$, $\sigma =\sqrt{\sigma_{𝐮}^{2}\sigma_{𝐯}^{2}}$, λ=$\frac{\sigma_{𝐮}}{\sigma_{𝐯}}$

Stage 3: The BCC model is re-applied using adjusted inputs and original outputs. This stage provides the final bias-corrected efficiency scores, which eliminate the influence of environmental variables and random noise.

### Dynamic analysis

The Malmquist Productivity Index was employed to characterize the dynamic evolution of hospital efficiency over the 2019–2023 period [38]:


$$\begin{array}{c} 𝐌({𝐱}_{𝐭+1},{𝐲}_{𝐭+1},{𝐱}_{𝐭},{𝐲}_{𝐭})=\frac{{𝐃}^{𝐭+1}({𝐱}_{𝐭+1},{𝐲}_{𝐭+1})}{{𝐃}^{𝐭}({𝐱}_{𝐭},{𝐲}_{𝐭})}\times \sqrt{\frac{{𝐃}^{𝐭}({𝐱}_{𝐭+1},{𝐲}_{𝐭+1})}{{𝐃}^{𝐭+1}({𝐱}_{𝐭+1},{𝐲}_{𝐭+1})}}\times \sqrt{\frac{{𝐃}^{𝐭}({𝐱}_{𝐭},{𝐲}_{𝐭})}{{𝐃}^{𝐭+1}({𝐱}_{𝐭},{𝐲}_{𝐭})}}. \end{array}$$ (9)

Total Factor Productivity (TFP) change was decomposed into Technological Change (TC) and Efficiency Change (EC) [39]. A TFP value greater than 1 signified productivity growth, while a value below 1 indicates a decline.

### Spatial autocorrelation

Spatial autocorrelation, a measure of spatial clustering of variables within a specific area, is divided into global and local forms. Moran's Index, a common tool for its assessment, statistically evaluates spatial correlations between neighboring regions [40, 41]. The global Moran's Index is used here to analyze the spatial dynamics of primary health care (PHC) systems across the entire study area, calculated as follows:


$$\begin{array}{c} 𝐈=\frac{\sum_{𝐢=1}^{𝐧}\sum_{𝐣=1}^{𝐧}{𝐖}_{𝐢𝐣}({𝐗}_{𝐢}-\overset{―}{𝐗})({𝐗}_{𝐢}-\overset{―}{𝐗})}{{𝐒}^{2}\sum_{𝐢=1}^{𝐧}\sum_{𝐣=1}^{𝐧}{𝐰}_{𝐢𝐣}}. \end{array}$$ (10)

X_i_ and X_j_ are the PHC service efficiency scores of cities i and j, respectively, S^2^ is the variance, and X is the mean of the efficiency scores. The global Moran's I ranges from −1–1. A value of 0 indicates no spatial autocorrelation, values near 1 show strong positive spatial autocorrelation (similar values cluster), and values near −1 indicate negative spatial autocorrelation (dissimilar values cluster). When I = 0, the spatial distribution is random.

The local Moran's I measures spatial clustering at the local level. It assesses spatial relationships between a city and its neighbors. Based on the city's efficiency and its neighbors’, the local Moran's I can identify four patterns:high-high, high-low, low-high, and low-low clusters [42]. The formulas are as follows.


$$\begin{array}{c} {𝐈}_{𝐢}=\frac{({𝐗}_{𝐢}-\overset{―}{𝐗})}{{𝐒}^{2}}\sum_{𝐣=1}^{𝐧}{𝐖}_{𝐢𝐣}({𝐗}_{𝐢}-\overset{―}{𝐗}). \end{array}$$ (11)

## Results

### Static efficiency analysis

Across the 31 provincial-level units, DEA results indicated a clear east-to-west gradient in efficiency. Four eastern regions—Beijing, Shanghai, Jiangsu, and Zhejiang—consistently remained on the production frontier (stage‑3 TE = 1.000) throughout the study period. In contrast, Qinghai (0.522) and Tibet (0.129) exhibited the largest input slacks in both bed capacity and health workforce. In terms of scale efficiency, approximately two‑thirds of provinces—including Fujian, Hainan, and Inner Mongolia—operated under increasing returns to scale, implying that marginal resource investments, when coupled with managerial improvements, could generate disproportionately higher service outputs [43] (Table 1).

**Table 1:** Efficiency of health resource allocation in 31 Chinese provinces.

Province	Stage1				Stage3			
	TE	PTE	SE	RTS	TE	PTE	SE	RTS
Anhui	0.799	0.87	0.918	drs	0.917	0.918	1	-
Beijing	1	1	1	-	0.811	0.923	0.879	irs
Fujian	0.838	0.843	0.993	irs	0.769	0.805	0.955	irs
Gansu	1	1	1	-	1	1	1	-
Guangdong	0.951	1	0.951	drs	0.999	1	0.999	drs
Guangxi	0.988	1	0.988	drs	1	1	1	-
Guizhou	0.914	0.922	0.991	drs	0.909	0.923	0.985	irs
Hainan	0.693	0.783	0.885	irs	0.503	0.761	0.662	irs
Hubei	0.948	1	0.948	drs	1	1	1	-
Henan	0.864	0.963	0.898	drs	0.917	0.991	0.925	drs
Heilongjiang	0.768	0.774	0.992	drs	0.959	1	0.959	irs
Hebei	0.731	0.747	0.979	drs	0.778	0.784	0.992	drs
Hunan	0.867	0.909	0.954	drs	0.89	0.908	0.98	drs
Jilin	0.9	0.932	0.966	irs	0.88	0.957	0.919	irs
Jiangsu	0.905	1	0.905	drs	1	1	1	-
Jiangxi	0.809	0.814	0.995	drs	0.816	0.826	0.988	irs
Liaoning	0.671	0.672	0.999	drs	0.69	0.716	0.964	irs
Inner Mongolia	0.662	0.672	0.986	irs	0.579	0.661	0.875	irs
Ningxia	0.935	1	0.935	irs	0.663	1	0.663	irs
Qinghai	0.724	0.895	0.809	irs	0.522	1	0.522	irs
Shandong	0.92	1	0.92	drs	0.951	1	0.951	drs
Shanxi	0.635	0.642	0.99	irs	0.624	0.683	0.913	irs
Shaanxi	0.851	0.855	0.995	drs	0.836	0.858	0.975	irs
Shanghai	1	1	1	-	1	1	1	-
Sichuan	0.929	1	0.929	drs	0.966	1	0.966	drs
Tianjin	0.903	1	0.903	irs	0.717	1	0.717	irs
Tibet	0.498	1	0.498	irs	0.129	0.757	0.17	irs
Xinjiang	1	1	1	-	0.919	0.987	0.931	irs
Yunnan	0.939	0.98	0.959	drs	0.982	0.984	0.998	irs
Zhejiang	1	1	1	-	1	1	1	-
Chongqing	0.957	0.963	0.993	drs	0.914	0.94	0.972	irs
mean	0.858	0.911	0.944		0.827	0.916	0.899	

### SFA regression analyses

The stochastic frontier regression modeled stage‑1 input slacks as the dependent variables [44]. Estimated coefficients revealed distinct environmental influences: urbanization rate showed positive associations with both personnel and bed slack, indicating an efficiency‑dampening effect. In contrast, per‑capita GDP exhibited negative coefficients, suggesting that higher regional economic development helps reduce resource waste. Urban population density had mixed effects: it decreased slack in bed utilization but slightly increased slack in health workforce, pointing to potential congestion diseconomies in highly dense metropolitan settings (Table 2). The likelihood‑ratio test strongly rejected the null hypothesis of no inefficiency (LR > 14, p < 0.01), confirming the appropriateness of the three‑stage modeling approach.

**Table 2:** SFA regression results.

	Number of health personnel	Number of beds	Number of health personnel
Cons	88435.98	111771.43	109580.32
GDP per capital	−18782.57	−49072.60	−43669.46
Urbanization rate	31517.20	83522.69	80353.80
Urban population density	−1419.49	10591.92	5021.93
$\sigma^{\text{2}}$	108312740.00	3698220800.00	3393769900.00
γ	1.00	1.00	1.00
LRtest	14.72	20.56	21.15

### Stage 3: DEA analysis again

**Fig 1:**
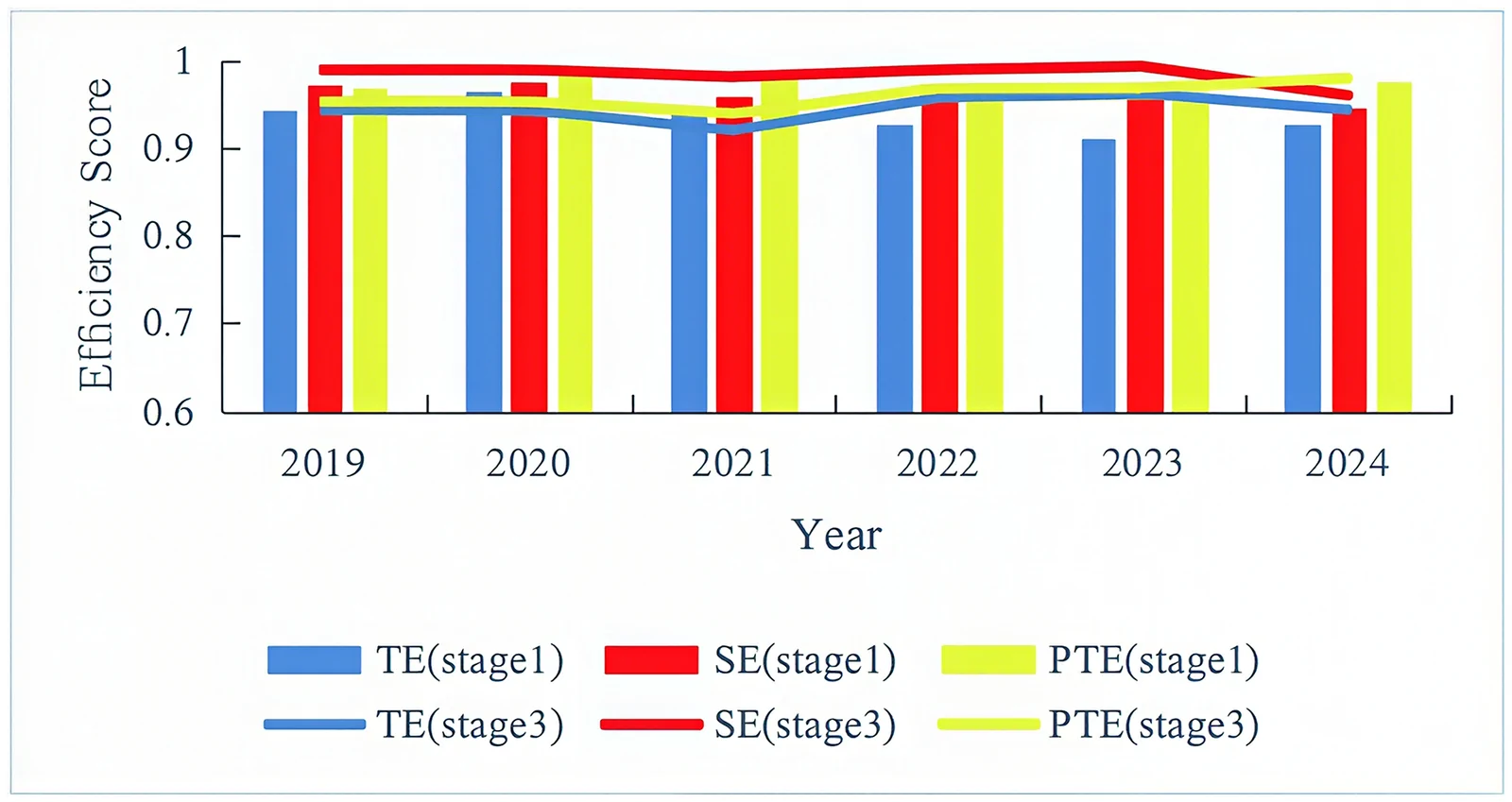
Comparison between stage 1 and stage 3 efficiencies (2019–2023).

The efficiency of healthcare resource allocation in Chinese hospitals is significantly influenced by external environmental factors. After correction via Stochastic Frontier Analysis (SFA), the national average comprehensive efficiency decreased from 0.858 to 0.827, and the average scale efficiency dropped from 0.944 to 0.899, indicating that the traditional DEA method may systematically overestimate efficiency levels due to uncontrolled environmental heterogeneity. In terms of regional performance, Gansu, Shanghai, and Zhejiang consistently remained on the DEA efficient frontier. The efficiency scores of 16 provinces, including Fujian and Beijing, declined after environmental adjustment, whereas the efficiency values of 12 provinces, such as Anhui and Guangdong, improved after excluding environmental factors (Table 1, Fig 1).

### Inter-provincial heterogeneity

**Fig 2:**
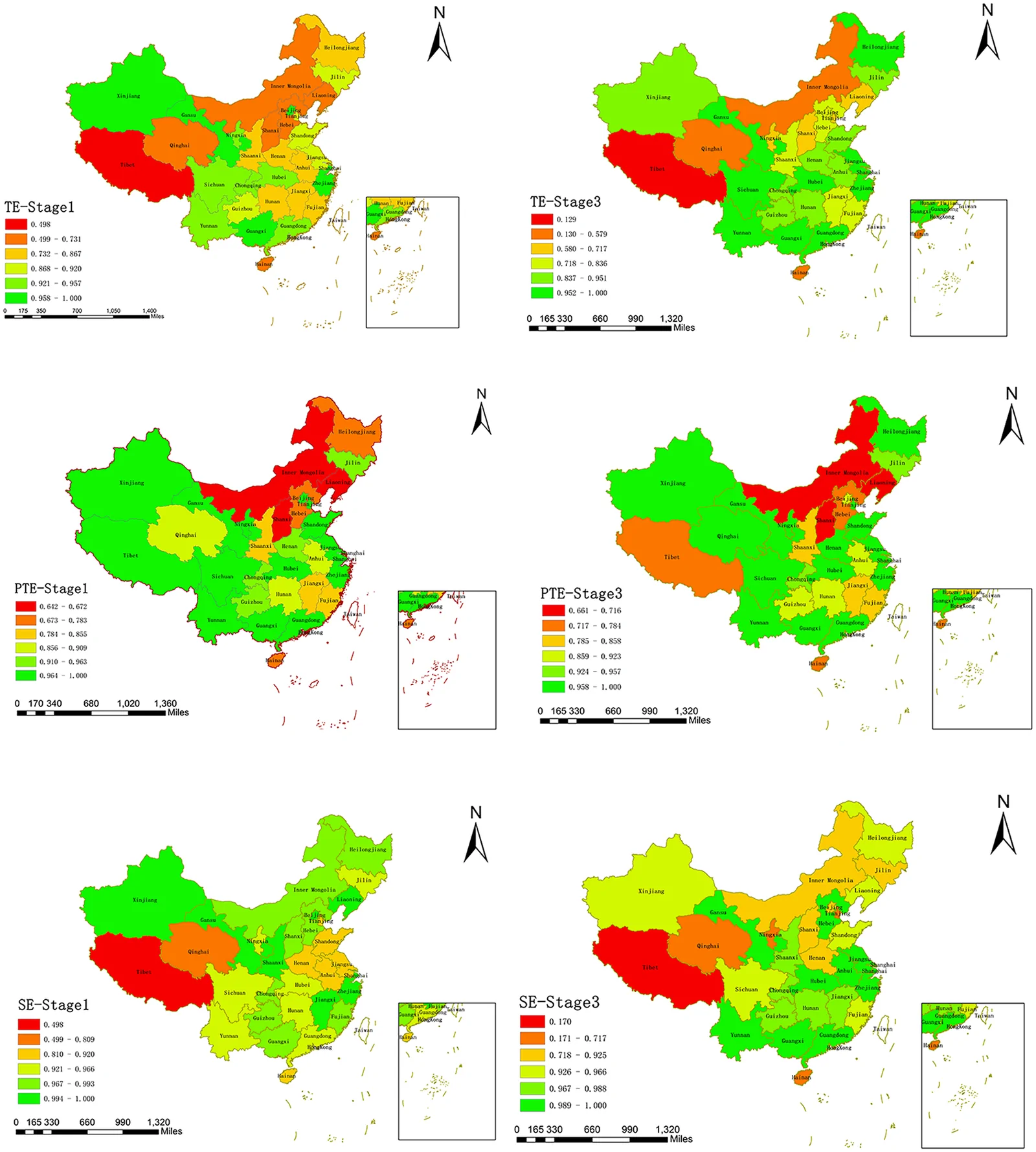
Comparison stage-1 and stage-3 efficiency in 31 provincial units.

Spatial analysis based on the third-stage efficiency scores reveals significant spatial heterogeneity in the efficiency of healthcare resource allocation in Chinese hospitals. A continuous high-efficiency corridor has formed in the middle and lower reaches of the Yangtze River, including Chongqing, Hunan, Jiangxi, Anhui, and Zhejiang, where efficiency values consistently exceed the national average. In contrast, the northeastern and northwestern peripheral provinces, such as Heilongjiang, Tibet, and Qinghai, constitute persistent low-efficiency cold spots. This spatial pattern aligns closely with soft infrastructure indicators, such as nighttime light intensity and high-speed rail network density [45], indicating that, in addition to hardware investments, soft conditions like market depth and transportation connectivity also profoundly influence the efficiency of resource allocation (Fig 2).

### Dynamic efficiency analysis

The overall efficiency of healthcare resource allocation in Chinese hospitals shows an upward trend, yet exhibits significant annual fluctuations and regional disparities. Total Factor Productivity (TFP) experienced two growth cycles in 2019–2020 (+3.2%) and 2020–2021 (+4.0%), followed by a sharp decline in 2021–2022 (−32.4%) due to the pandemic and stringent mobility restrictions. As the pandemic eased and backlogged medical services gradually resumed, TFP rebounded strongly in 2022–2023 (+88.2%). This substantial growth, calculated using bias-corrected Stage-3 data, is a statistically robust reflection of the “V-shaped” recovery from the low adjusted base in 2022 (TFP = 0.676). The magnitude reflects the sudden release of accumulated medical demand after the lifting of mobility restrictions, rather than a steady-state annual trend. From 2021 to 2023, the trend of Technological Change (TC) aligned with that of the TFP index, with its growth magnitude far exceeding that of Efficiency Change (EC), indicating that the primary driver of the recovery was advancements at the technological frontier, such as telemedicine and day surgery, rather than catch-up improvements in managerial efficiency (Table 3).

**Table 3:** Dynamics of efficiency of PHC services (2019–2023).

Year	EC	TC	PECT	SEC	TFP
2019-2020	1.013	1.018	0.903	1.121	1.032
2020-2021	1.009	1.031	1.004	1.005	1.04
2021-2022	0.851	0.794	1.058	0.805	0.676
2022-2023	1.217	1.546	0.964	1.263	1.882
Mean	1.014	1.066	0.98	1.035	1.081
F-statistic	6.92	10.34	1.45	5.78	8.47
P-value	0.021*	0.008*	0.316	0.032*	0.013*

Note: *:p < 0.05;**:p < 0.01.

### Spatial autocorrelation analysis

#### Global autocorrelation.

The Global Moran’s I index fluctuated between 0.266 and 0.308 (all p-values < 0.01), confirming the presence of significant positive spatial dependence in efficiency—high-efficiency provinces exhibited spatial clustering rather than random dispersion. The index peaked in 2020 (0.308), likely due to enhanced cross-provincial policy coordination during the COVID-19 pandemic, and subsequently declined to 0.299 in 2023 as regional response strategies diverged (Table 4).

**Table 4:** Global Moran’s I Index for 2019-2023.

Year	Moran's I	P
2019	0.266	0.005
2020	0.308	0.002
2021	0.303	0.002
2022	0.289	0.003
2023	0.299	0.001

### Local autocorrelation

**Fig 3:**
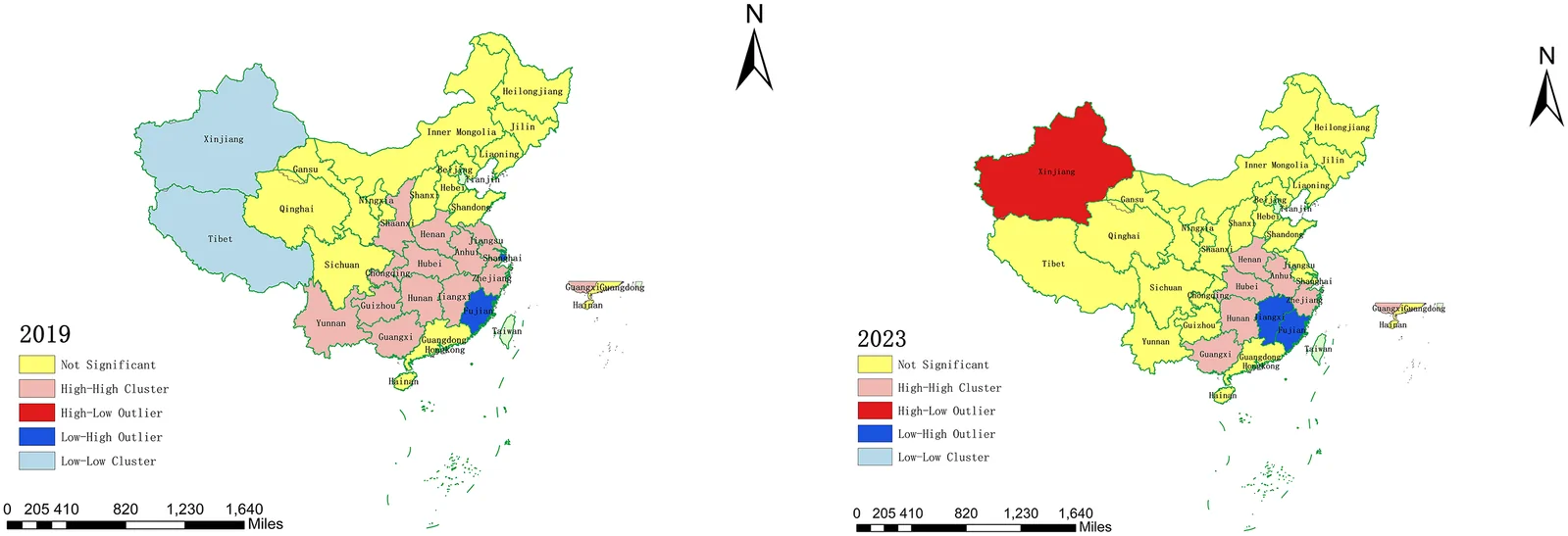
Local Indicators of Spatial Association (LISA) map of Service efficiency.

Between 2019 and 2023, the spatial distribution of hospital-based health resources in China showed a trend of optimization and consolidation. By 2023, seven provinces— Guangxi, Hunan, Hubei, Henan, Anhui, and Zhejiang—displayed a High-High clustering pattern, forming a contiguous efficiency radiation belt concentrated in the Central and South-Central regions. Notably, the Low-Low cluster consisting of Xinjiang and Tibet in 2019 had completely disappeared by 2023, reflecting a significant catch-up effect in these western regions. Xinjiang shifted from a Low-Low cluster to a High-Low outlier, indicating its emergence as an isolated efficiency island. Meanwhile, the Low-High outliers increased from one (Fujian) to two (Fujian and Zhejiang), suggesting that while these eastern provinces are surrounded by high-efficiency neighbors, their internal operational processes still have room for further alignment with the regional frontier (Fig. 3).

### Overall efficiency analysis

The efficiency of healthcare resource allocation in Chinese hospitals exhibits a distinct regional gradient, ranked in the order of eastern > central > western regions. In 2023, the average comprehensive efficiency was 0.934 in the eastern region, 0.932 in the central region, and 0.902 in the western region, showing a stepwise decline. Both pure technical efficiency and scale efficiency exceeded 0.9 across all regions, with no significant cross-regional disparities. After adjusting for environmental factors, the central region surpassed the eastern region in comprehensive efficiency, pure technical efficiency, and scale efficiency, indicating that the regional gradient is not solely attributable to differences in hardware inputs but is more strongly influenced by external soft environment factors (Table 5).

**Table 5:** Comparison of regional health resource allocation efficiency in China.

Area	Stage1			Stage3		
	TE	PTE	SE	TE	PTE	SE
East	0.934	0.972	0.961	0.875	0.973	0.9
Central	0.932	0.988	0.942	0.945	0.985	0.957
West	0.902	0.952	0.947	0.835	0.937	0.883

## Discussion

This study contributes a bias-corrected, full-cycle (2019–2023) evaluation of hospital efficiency in China by integrating three-stage DEA with spatial autocorrelation, revealing that environmental dividends mask the Central region’s higher latent governance resilience and that inter-provincial efficiency converges with persistent high-high clustering south of the Yangtze River (see S1 Table in S1 Appendix for a summary of key findings).

Hospital inefficiency remains a major constraint for low- and middle-income countries in achieving universal health coverage [46]. Evidence from Asian health systems indicates that an average of 27% of inputs in public hospitals could be saved without affecting outputs [47]. The results of this study show that the national average comprehensive efficiency of hospitals is only 0.827, reflecting an overall low efficiency in resource allocation. Among them, 83.9% of provinces have not reached optimal efficiency, and 51.6% of provinces exhibit decreasing returns to scale, with no significant improvement over time, indicating that further increases in inputs will yield proportionally smaller output gains [48]. For every 1,000 yuan increase in per capita GDP, input redundancy decreases by 4.9%, whereas a 1 percentage point increase in urbanization rate expands redundancy by 3.1%. The inhibitory effect of urbanization rate and urban population density on efficiency aligns with findings from previous studies, which highlight congestion effects in rapidly urbanizing provinces [49]. After removing the influence of environmental factors, central provinces simultaneously achieved improvements in both PTE and SE in the third stage, temporarily shifting the regional efficiency gradient from eastern > central > western to central > eastern > western [50, 51]. In terms of spatial distribution, the Moran’s I index is greater than 0 with a P-value below 0.05, indicating a positive spatial correlation in the efficiency of health resource allocation in Chinese hospitals. A stable high-efficiency cluster persists in the region south of the Yangtze River, while low-efficiency clusters had largely dissipated by 2023, reflecting a convergence trend of provincial efficiency levels toward the efficiency frontier.

After removing the influence of environmental factors, the average efficiency of healthcare resource allocation in hospitals in the central region surpassed that of the eastern region. Third-stage results show that the central region achieved the highest comprehensive efficiency (0.945), followed by the eastern region (0.875) and the western region (0.835). This finding suggests that the efficiency advantage of the eastern region primarily relied on its higher market demand density, high-speed rail network connectivity, and fiscal capacity, rather than absolute superiority in hospital governance [52]. Once these external environmental advantages were stripped away, internal managerial capabilities—such as lean process reengineering, implementation of hierarchical medical systems, and flexibility in resource allocation—emerged as the dominant determinants of efficiency, thereby revealing the latent organizational and operational advantages of the central region [53].

Technical efficiency remains the primary constraint on total factor productivity. Decomposition of the Malmquist index based on stochastic frontier analysis indicates that the 88.2% rebound in total factor productivity (TFP) from 2022 to 2023 was predominantly driven by technological change. Over the entire study period, technological change exhibited an average annual growth rate of 6.6%, far exceeding the 1.4% annual improvement in efficiency change, while scale efficiency change remained statistically stable. This divergence suggests that the adoption of advanced technologies such as tele-consultation, day-surgery bundles, and AI-assisted imaging has shifted the production frontier outward, whereas improvements in managerial efficiency have lagged. Furthermore, provinces with persistently low technological change, such as Tibet and Qinghai, also demonstrated the slowest efficiency recovery, indicating that regions lagging in technology often simultaneously face organizational rigidities [54]. Therefore, policy interventions targeting pure technical efficiency—such as clinical pathway redesign, performance-based contracting, and digital triage—yield greater marginal returns to TFP compared to simply expanding bed capacity or increasing service reimbursement coverage.

From 2019 to 2023, the efficiency of healthcare resource allocation in Chinese hospitals exhibited a significant trend of inter-provincial convergence. The Global Moran’s I index remained consistently above 0.293 (p < 0.01), indicating stable positive spatial autocorrelation in hospital resource efficiency. Local Indicators of Spatial Association (LISA) further identified a persistent high-high cluster in the region south of the Yangtze River, which expanded from four provinces in 2019 to seven provinces in 2023, forming a contiguous belt. In contrast, the low-low cluster had completely disappeared by 2023, and the number of low-high outliers decreased from three to two. The β-convergence coefficient revealed an annual efficiency catch-up rate of 4.6%, suggesting that inter-provincial spillover effects—facilitated by mechanisms such as telemedicine platforms, cross-provincial direct settlement, and specialist tele-rounds—are effectively promoting the diffusion of managerial experience and technological knowledge [55]. As the externalities of soft infrastructure gradually become internalized, the efficiency frontier continues to expand outward, and inter-provincial disparities steadily narrow. This provides empirical support for advancing regionally integrated health system reforms, rather than relying solely on isolated facility-level investments.

The findings indicate that expanding inputs alone is unlikely to improve hospital efficiency in 51.6% of provinces; managers should instead focus on resource reallocation and lean operations. Eastern hospitals need internal governance reforms rather than relying on favorable economic conditions. Finally, technological investments must be paired with management training to convert adoption into productivity gains.

## Conclusion

Based on provincial panel data from 2019 to 2023, this study systematically evaluates the efficiency of healthcare resource allocation in Chinese hospitals, yielding the following main conclusions: First, there remains significant room for improvement in overall efficiency, with more than half of the provinces exhibiting decreasing returns to scale. This suggests that continued expansion of factor inputs alone may not yield proportional output gains, and structural optimization deserves greater attention.. Second, environmental factors significantly interfere with efficiency assessments. After controlling for external variables such as per capita GDP and urbanization rate, the regional efficiency ranking from the conventional pattern of eastern > central > western to central > eastern > western, indicating that observed regional differences are partly attributable to environmental heterogeneity rather than intrinsic operational performance alone. Third, total factor productivity growth is primarily driven by technological change, while efficiency change lags considerably. This suggests that the adoption of new technologies has advanced the production frontier, yet further gains could be achieved by addressing underlying inefficiencies in resource utilization. Fourth, hospital efficiency demonstrates significant positive spatial correlation, with provincial efficiency levels continuously converging toward the production frontier. This pattern is consistent with the influence of regional coordination mechanisms and technology diffusion across provinces.

This study has several limitations. including the use of provincial-level aggregated data, unmeasured confounding factors, and potential endogeneity among variables. Future research incorporating facility-level information and dynamic panel models could address these issues.

## Supporting information

S1 Appendix Integrated three-stage DEA-malmquist-spatial analysis framework, including the model framework (S1 Fig) and key findings summary (S1 Table). (DOCX)
