# Supplementary material for: A study on the efficiency and spatial correlation of health resource allocation in Chinese hospitals
Source: PLoS One. 2026 Sep 8;21(9):e0356197. doi: 10.1371/journal.pone.0356197 (PMC13552783; doi:10.1371/journal.pone.0356197)
Supplement: Supplementary file 1 [file pone.0356197.s001.docx]

Supplementary Materials

**S1 Text. Integrated Three ‑ stage DEA ‑ Malmquist‑Spatial Analysis Framework**

**Input:** the number of medical institutions, hospital beds, health technicians

**Output:** outpatient visits, inpatient discharges

**Stage 1: Initial Efficiency Evaluation (DEA - BCC Model)**

(1) For each province i in year t:

(2) Solve the input-oriented BCC model to obtain efficiency scores (TE, PTE, SE) and input slacks (S⁻).

**Stage 2: Environmental Adjustment (SFA Regression)**

(1) Regress input slacks S_it_ against environmental variables Z_it_ using the SFA model: $S_{ni}$=f($Z_{ni}$; $\beta$)+$v_{ni}$+$u_{ni}$.
(2) Adjust raw inputs to eliminate environmental effects and random noise, ensuring all units are compared under a standardized environment: $\text{X}_{\mathbf{ni}}^{\text{A}}$**=**$\text{X}_{\mathbf{it}}$**+[max(f(**$\text{Z}_{\mathbf{ni}}$**;**$\text{\textasciicircumβ}_{\text{n}}$**))-f(**$\text{Z}_{\mathbf{ni}}$**;**$\text{\textasciicircumβ}_{\text{n}}$**)]+[max(**$\text{v}_{\mathbf{ni}}$**)-**$\text{v}_{\mathbf{ni}}$**].**

**Stage 3: Bias-Corrected Efficiency Re-measurement (DEA)**

(1) Re-run BCC model using (x_adj_) and original outputs to obtain final scores.

**Dynamic Analysis: Malmquist Productivity Index**

(1) Calculate Malmquist Index: (TFP = EC × TC）.
(2) Calculate Global and Local Moran’s I for spatial clustering.

**Spatial Analysis: Autocorrelation and Clustering**

Compute Global Moran's I to test for overall spatial autocorrelation, and Local Moran's I (LISA) to identify High-High, High-Low, Low-High, and Low-Low clustering patterns.

Fig S1. Logical Framework of the Three - stage DEA Model

Table S1. Summary of key findings on hospital efficiency in China (2019–2023)

| Dimension | Key Indicator | Core Finding / Value |
| --- | --- | --- |
| Static Efficiency | National Average Comprehensive Efficiency | Stage 1: 0.858 → Stage 3: 0.827 (Bias-corrected) |
| Regional Ranking | Efficiency Gradient (Post-adjustment) | Central (0.945) > East (0.875) > West (0.835) |
| Dynamic Efficiency | Mean Total Factor Productivity (TFP) | 1.081 (Primarily driven by Technological Change) |
| Spatial Correlation | Global Moran’s I Index | 0.266 – 0.308 (Significant positive autocorrelation) |
| Spatial Patterns | High-High Clustering | Stable south of the Yangtze River; expanded to 6 provinces by 2023 |
| Returns to Scale | Dominant State | 51.6% of provinces exhibit decreasing returns to scale |
